# The role of nociceptive neurons in allergic rhinitis

**DOI:** 10.3389/fimmu.2024.1430760

**Published:** 2024-08-09

**Authors:** Jianchao Cong, Hao Lv, Yu Xu

**Affiliations:** ^1^ Department of Otolaryngology-Head and Neck Surgery, Renmin Hospital of Wuhan University, Wuhan, China; ^2^ Department of Rhinology and Allergy, Renmin Hospital of Wuhan University, Wuhan, China; ^3^ Research Institute of Otolaryngology-Head and Neck Surgery, Renmin Hospital of Wuhan University, Wuhan, China; ^4^ Hubei Province Key Laboratory of Allergy and Immunology, Wuhan, China

**Keywords:** allergic rhinitis, nociceptive neurons, nociceptive neuron receptors, neuropeptides, immune system

## Abstract

Allergic rhinitis (AR) is a chronic, non-infectious condition affecting the nasal mucosa, primarily mediated mainly by IgE. Recent studies reveal that AR is intricately associated not only with type 2 immunity but also with neuroimmunity. Nociceptive neurons, a subset of primary sensory neurons, are pivotal in detecting external nociceptive stimuli and modulating immune responses. This review examines nociceptive neuron receptors and elucidates how neuropeptides released by these neurons impact the immune system. Additionally, we summarize the role of immune cells and inflammatory mediators on nociceptive neurons. A comprehensive understanding of the dynamic interplay between nociceptive neurons and the immune system augments our understanding of the neuroimmune mechanisms underlying AR, thereby opening novel avenues for AR treatment modalities.

## 1 Introduction

Allergic rhinitis (AR) is a chronic nasal mucosa disorder mediated by immunoglobulin E (IgE) in response to allergen exposure, eliciting a predominantly Th2-type immune response. Key symptoms encompass a runny nose, nasal congestion, nasal itching and sneezing. The global prevalence of AR has risen steadily in recent years, currently impacting approximately 10-40% of the world’s population, thus ranking among the most common chronic ailments. AR exerts a significant physical and mental burden on patients, impacting their academic, professional and lives (1). While available treatments, including medications and immunotherapy, can alleviate symptoms, a definitive cure for AR remains elusive. The immune theory alone does not fully reveal the pathological mechanism of AR. Apart from the immune system, the nervous system also plays a crucial role in AR pathogenesis (2). The nasal mucosa boasts a complex nervous system, wherein sensory nerves serve as afferent nerves, predominantly responding to external stimuli. Sensory neurons within the nasal mucosa encompass nociceptive neurons, perceiving nociceptive stimuli, and non-nociceptive neurons sensing general and special stimuli like temperature, touch and olfaction. Nociceptive neurons, specifically, detect various harmful stimuli such as mechanical injury, chemicals, inflammatory mediators and pathogens. These neurons transmit information about noxious stimuli to the brain and release neuropeptides from nerve endings, influencing immune cells and modulating local immune responses (3). Conversely, mediators released during inflammation can reciprocally regulate nociceptive neuron function (4).

A comprehensive understanding of the role of nociceptive neurons in AR provides deeper insights into the neuroimmune aspects of AR pathogenesis, offering novel perspectives for AR treatment strategies. This review delves into the anatomical distribution and functional characteristics of nociceptive neurons in the nasal mucosa, focusing on the interaction between nociceptive neurons and the immune system in AR.

## 2 The concept of nociceptive neurons and their anatomy in the nasal mucosa

**Figure 1:**
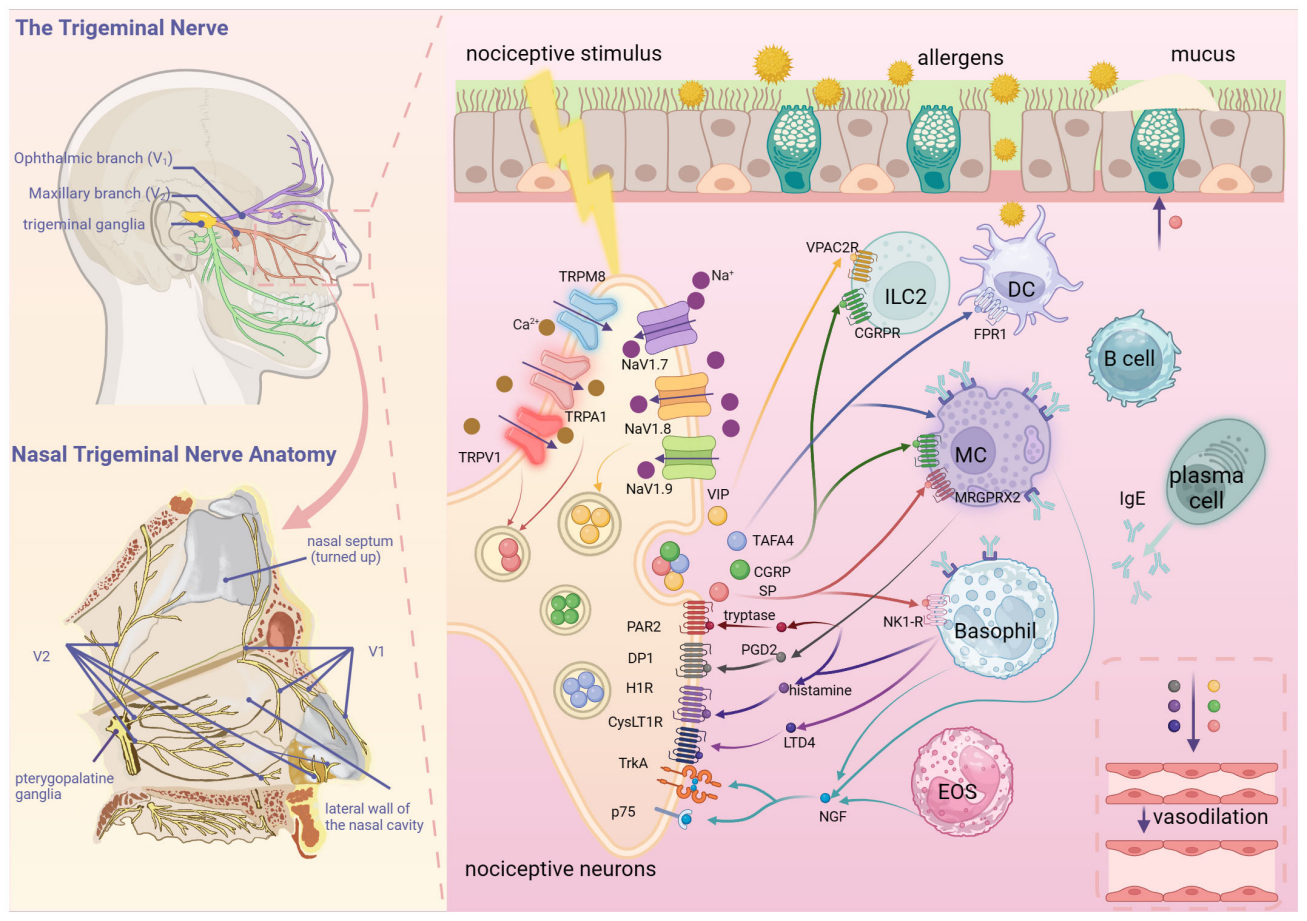
Nociceptive neuron-immune cell interaction network in allergic rhinitis. Nociceptive neurons in the nasal mucosa mainly originate from V1 and V2 of the trigeminal nerve. A variety of nociceptive stimuli, such as mechanical injury, chemicals, inflammatory mediators and pathogens, can activate nociceptive neurons by acting on TRPV1, TRPA1 and other receptors. Nociceptive neurons release neuropeptides, such as SP and CGRP, to induce the secretion of nasal glands, vasodilatation and activation of immune cells. Under the influence of allergen stimulation and neuropeptides, immune cells such as mast cells and basophils release inflammatory mediators such as histamine and cytokines, as well as nerve growth factors to increase the excitability of nociceptive neurons, resulting in neuroimmune circulatory pathway formation. (Created with BioRender.com).

Sensory neurons, encompassing nociceptive and non-nociceptive neurons, transform diverse stimuli into nerve impulses. Nociceptive neurons, primary sensory neurons, sense and transmit a spectrum of noxious stimuli, influencing inflammatory responses and maintaining bodily homeostasis. The trigeminal nerve sends the ophthalmic (V1), maxillary (V2) and mandibular branches forward from the trigeminal ganglion (TG). The ophthalmic nerve sends branches via the supraorbital fissure out of the cranium, which primarily supply the anterior and superior portions of the nasal cavity, while the maxillary nerve sends branches via the pterygopalatine fossa, which primarily supply the inferior and posterior portions of the nasal cavity, innervating the nasal mucosa’s general sensation (**Figure 1**). Comprising mainly small, nociceptive neurons containing multiple peptides, the TG forms unmyelinated, naked nerve endings intricately distributed in nasal vessels, glands and epithelium (5). Upon activation, nociceptive neurons transmit signals generated by these primary afferent nerves to the central nervous system, eliciting sensations like pain, itching and reflexes such as coughing or sneezing and activating preganglionic autonomic neurons, which initiate sympathetic and parasympathetic reflexes. Thus, the activation of nociceptive neurons produces solid sensations and induces protective reflexes. Allergenic excitation has often been associated with increased activation of afferent nerve endings, leading to action potential discharges. Moreover, nociceptive neuronal afferent fibers are a type of afferent fibers susceptible to direct activation by allergic mediators. Allergic mediators may overexcite nociceptive neurons to the extent that subthreshold stimuli or even non-nociceptive conventional stimuli may induce nociceptor-associated reflexes (6).

The afferent fibers of sensory neurons consist of myelinated Aα fibers (large fibers responsible for proprioception and motor control), Aβ fibers (large fibers that transmit touch and pressure sensations), Aδ fibers (small fibers that transmit sharp pain sensations) and unmyelinated C fibers (small fibers that transmit dull, aching pain and some temperature sensations). Nociceptive neurons in the nasal mucosa primarily involve myelinated Aδ fibers for immediate pain and unmyelinated C fibers for delayed-onset chronic pain (2). These C-fiber-mediated nociceptions can be increased when subjected to repeated chemical stimuli (7). Functionally, Aδ fibers primarily respond to nociceptive mechanical stimuli and pain and itching sensations. Moreover, nociceptive C fibers responding to multiple stimuli can transmit various sensations such as burning, stinging, warmth, cold and itching. Importantly, various nociceptive stimuli and inflammatory mediators such as capsaicin, heat, H ^+^, K ^+^, mechanical stimuli, bradykinin (BK) and TNF-α can activate C-fiber nociceptive neurons (8, 9). Studies reveal that pathogenic organisms, such as bacteria, viruses and worms, can induce airway inflammation by directly acting on nociceptive sensory neurons in the trigeminal nerve (10, 11). Different types of nociceptive stimuli may activate different subpopulations of nasal C-fiber nociceptive neurons, inducing a protective response in the body by increasing nasal airflow resistance (12). Following a nociceptive stimulus, peripheral nociceptive sensory nerve endings activate and release a variety of neuropeptides that induce plasma extravasation, promote immune cell migration and mediate the inflammatory response, a phenomenon which is known as neurogenic inflammation (13, 14). Several markers of nociceptive neurons, including Sodium voltage-gated channel alpha subunit 10 (SCN10A), Potassium channel subfamily K member 18 (KCNK18) and MAS-related G protein-coupled receptor D (MRGPRD), play roles in nociceptive sensation with high TG specificity (15, 16). Notably, SCN10A is specifically associated with inflammatory pain (17). Moreover, KCNK18 mRNA is expressed only in human TG and is closely associated with migraine (18), whereas MRGPRD specifically expresses on nociceptors in human TG and dorsal root ganglion (DRG), which are involved in the perception and modulation of nociception (19). Expression of SCN10A (20), KCNK18 and MRGPRD has been detected in the nasal mucosa. However, nociceptive neurons in the nasal mucosa cannot be quantified by subtype due to the lack of specificity of the marker genes (21). Additionally, protein gene product 9.5 (PGP 9.5) and microtubule-associated protein 2 (MAP2) serve as general neuronal markers. Detection of these markers contributes to a nuanced understanding of nociceptive neuron functional changes.

## 3 Role of nociceptive neuronal receptors in AR

Nociceptive neurons express various receptors, such as transient receptor potential (TRP) receptors, voltage-gated sodium channels (NaVs) and toll-like receptors, positioned at peripheral nerve terminals. These molecular sensors empower nociceptive neurons to detect various environmental threats, playing a significant role in AR.

### 3.1 Transient receptor potential

TRP receptors, non-selective cation channel proteins that can be activated by a variety of stimuli, are responsible for a wide range of sensory responses including heat, cold, pain, stress, vision and taste (22). Predominant in nociceptive neurons (23), TRP receptors are a diverse group with six subgroups: classical transient receptor potential (TRPC), transient receptor potential vanilloid (TRPV), transient receptor potential ankyrin (TRPA), M-type transient receptor potential (TRPM), polycystin-like transient receptor potential (TRPP) and mucolipid-like transient receptor potential (TRPML) (24). These receptors detect stimuli such as heat, cold, acidosis, osmolarity and environmental factors, activating Ca^2+^ influx upon stimulation. This influx leads to neuronal depolarization, generating action potentials that transmit nociceptive signals to the CNS and triggers reflexes such as coughing and sneezing (25). Sensitization and activation of TRP receptors involve multiple protein kinases, such as protein kinase C, protein kinase A and Ca^2+^/calmodulin-dependent kinase II (26, 27). TRPV1, TRPA1 and TRPM8 are expressed in the human nasal mucosa (28, 29). Studies on TRP receptors in AR primarily focus on TRPV1, TRPA1and TRPM8.

TRPV1, primarily expressed on nociceptive neurons, plays a significant role in AR pathogenesis and symptoms. *In vivo*, neuronal tracer methods have demonstrated that nasal mucosal sensory neurons respond to capsaicin and generate action potentials; however, this effect was inhibited by a selective inhibitor of TRPV1, thereby confirming the presence of TRPV1 on nasal mucosal sensory neurons (30). During seasonal allergen exposure, patients with allergic rhinitis feature an increased itch response to TRPV1 stimulation (31). Activation of TRPV1 by nociceptive stimuli leads to the release of pro-inflammatory mediators, manifesting as AR symptoms such as sneezing, runny nose, itchy nose and nasal congestion (30). Capsaicin, a significant TRPV1 agonist, activates nociceptive neurons (32). For instance, an early clinical study found that high-dose capsaicin stimulation of the nasal mucosa in patients with AR promoted nasal glandular secretion and plasma extravasation (33). Additionally, TRPV1^+^ peptidergic nerve fibers and mucin-5 subtype B (MUC5B)^+^ submucosal glands are anatomically adjacent. Studies have demonstrated that MUC5B levels were increased in nasal lavage fluids after nasal stimulation with capsaicin, suggesting that TRPV1 activation on sensory nerve fibers is likely to promote MUC5B release from submucosal glands (34). Thus, targeting TRPV1 emerges as a potential strategy for AR treatment. However, the prolonged application of capsaicin leads to the desensitization of nociceptive neurons (35) and the alleviation of neurogenic inflammation in AR, possibly by blocking the axon reflex and neuropeptides secreted by nociceptive neurons, thereby preventing them from responding to further stimuli (8, 36). The efficacy of capsaicin in AR treatment remains unclear, possibly influenced by dosage, treatment duration and AR pathogenesis complexity. Azelastine hydrochloride (AZE) is an intranasal antihistamine and fluticasone propionate (FP) is an intranasal corticosteroid commonly used to relieve the symptoms of allergic rhinitis (37). *In vitro*, studies have demonstrated that repeated application of AZE + FP significantly induces desensitization of TRPV1^+^ sensory neurons and that this desensitization may be due to strong Ca2+ influx and inability to translocate neurotrophic factors as a result of neuronal excitation (38). Additionally, the selective TRPV1 antagonist SB-705498 was demonstrated to alleviate AR by blocking TRPV1 in a guinea pig model (30). Similarly, in patients with AR, nasal irrigation of SB-705498 improved capsaicin-induced nasal symptoms. It is also worth mentioning that applications of this TRPV1 antagonist had no effects on symptom score in allergen-challenged patients with allergic rhinitis. Additional studies should then be conducted to investigate the role of TRPV1 as a drug target in allergic rhinitis using longer-acting drug formulations. The sex-related differences concerning AR are still unclear. However, studies have shown that the incidence of AR is higher in females (39). A clinical study demonstrates that allergic rhinitis in adulthood may be associated with elevated levels of estrogen early in ontogenetic development (40). Recent research highlights the influence of estrogen on TRPV1 expression levels. Estrogen stimulates prolactin, promoting phosphorylation of TRPV1 and lowering its activation threshold (41). This effect of estrogen on TRP potentially contributes to the development of allergic diseases in mature women.

In addition to TRPV1, TRPA1 on nociceptive neurons also exerts a significant influence on AR. In the upper airway, TRPA1 is primarily expressed on nociceptive C fibers and responds to various external chemicals (alcohols, H_2_O_2_, NO, ozone and LPS), intense cold sensations and endogenous substances produced during tissue injury. Many TRPA1^+^ nociceptive neurons co-express TRPV1 and can be activated or sensitized by an elevation in intracellular Ca^2+^ (42). Blocking TRPA1 in trigeminal sensory neurons reduces the inflammatory response to allergen provocation and decreases the activity of nociceptive neurons (43). TRPA1 knockout mice exhibit decreased allergen-induced immune cell infiltration, lower pro-inflammatory cytokine expression levels and reduced hyperresponsiveness to nociceptive stimuli (44). Administration of a TRPA1 inhibitor significantly curtails the upregulation in the number of TRPA1^+^ nociceptive neurons in AR mice. Consequently, this downregulates airway Substance P (SP) levels, blocks the TRPA1-SP inflammatory pathway and improves Th2-type inflammation and nose-swabbing behaviors. Moreover, downregulating TRPA1 significantly reduced leukocyte counts and IL-8 levels in alveolar lavage fluid, inhibited lower airway remodeling and fibrosis and attenuated airway hyperresponsiveness (45). Therefore, targeting TRPA1 emerges as a potential therapeutic strategy to alleviate AR inflammation and nasal hyperreactivity (NHR). Additionally, TRPA1 is proposed as the primary oxidant sensor in nasal nociceptive neurons, wherein oxidants induce TRPA1-dependent Ca^2+^ influx, activating nasal nociceptive neurons and exacerbating AR (46).

TRPM8, expressed mainly on nociceptive sensory neurons, functions as a receptor sensitive to cold and menthol stimuli, earning it the nickname “cold receptor” and “menthol receptor”. Morphological experiments have demonstrated that TRPM8, expressed on trigeminal afferent fibers, is predominantly located in the perivascular area of the nasal mucosa. This potentially mediates neurovascular reflexes and is associated with runny nose symptoms (28, 43, 47). NHR is prevalent in all chronic upper airway inflammatory phenotypes, including AR. A recent study found that the expression of TRPM8 in nasal mucosa, a neuronal marker, correlates with NHR in AR patients (48). Persistent cough is a common symptom of upper airway diseases, including AR. Menthol, muscimol or camphor are main TRPM8 agonists. In animal models of NHR, nasal administration of camphor and muscimol reduced the number of coughs induced by citric acid or capsaicin. Furthermore, the intranasal application of menthol reduced the sensation of airway irritation, modulated the cough threshold and reduced the number of coughs (49). These findings suggest that TRPM8 agonists may increase the cough threshold and alleviate cough symptoms in AR by acting on TRPM8 in the nociceptive neurons of the nasal mucosa. In conclusion, TRPM8 plays a significant role in neuroimmunity in allergic diseases, but further exploration is needed to understand the involvement of nociceptive neuronal TRPM8 in AR.

### 3.2 Other receptors

In addition to TRP, nociceptive neurons express various receptors that contribute to the pathogenesis of AR. Voltage-gated sodium channels (NaVs), a family of ionotropic receptors abundant in nociceptive neurons, encompass nine members (NaV1.1-1.9 9) playing crucial roles in transmitting signals related to chronic pain and pruritus (50). Nav1.7, Nav1.8 and Nav1.9 are mainly expressed in sensory neurons, including nociceptors (51). Neuronal hypersensitivity is when a neuron responds in an abnormally strong manner to normal or mild stimuli and causes pain or discomfort. These channels, specifically Nav1.7-1.9, are implicated in neural action potentials and are associated with neuronal hypersensitivity. In AR, there is an observed increase in the number of Nav1.7-1.9^+^ nerve fibers (20). Inflammatory mediators linked to AR, such as NGF and nitric oxide (NO), upregulate the expression of Nav1.7-1.9^+^ nerve fibers (43). Notably, inhibiting Nav1.8^+^ nociceptive neurons with QX-314, a charged sodium channel inhibitor that enters via large-pore ion channels to specifically block nociceptors, significantly reduced allergic airway inflammation induced by OVA or dust mites (52). However, the specific role of NaV1.7 and Nav1.9 in allergic rhinitis remains to be investigated.

Toll-like receptors (TLRs), crucial pattern recognition receptors in neuroimmunity, are expressed by nociceptive neurons. This includes TLR2, which recognizes bacterial lipoproteins, TLR4, which recognizes lipopolysaccharides (LPS), and TLR5, which recognizes flagellin (25). Synthetic bacterial lipopeptides, which act as TLR2 agonists, have been demonstrated to ameliorate allergic airway inflammation by modulating the Th1 and Th2 responses (53). LPS, a classical TLR4 agonist, has been demonstrated to directly activate nociceptors through TLR4, resulting in TRPV1 sensitization, calcium influx and calcitonin gene-related peptide (CGRP) release (54, 55). Furthermore, LPS has been demonstrated to mediate allergic rhinitis through the TLR4/MyD88 pathway (56). Flagellin from gram-negative bacteria can bind to TLR5 on nociceptive neurons that mediate neuropathic pain (57). Evidence supports the co-localization of TLRs and TRP receptors on nociceptive neurons, indicating interactions such as TLR4 and TRPV1 (58), TLR7 and TRPA1 (59). TLR activation sensitizes TRP receptors, leading to the release of SP (54) and potentially exacerbating nasal neurogenic inflammation. Additionally, binding of related ligands to TLR7 promotes SP release from nociceptive neurons and mediates nasal neurogenic inflammation (60), potentially through the myeloid differentiation factor 88 (MyD88) pathway (61). Stimulation with the TLR7 agonist R-837 and the TLR7/8 agonist R-848 resulted in a rapid release of SP from sensory neurons (60).

Nociceptive neurons also express the Fc receptor (FcR) (62, 63), a class of cell surface proteins that bind specifically to the Fc segment of immunoglobulins, playing a crucial role in antibody-dependent immune responses. Allergen-IgE immune complexes (ICs) act on nociceptive neurons expressing FcϵRI, promoting Ca^2+^ influx through the FcϵRI-TRPC3 axis. This activation induced SP release, initiating Th2 cell activity, leading to the production of IL-5 and IL-13 and consequently triggering allergic airway inflammation (64).

Mas-related G protein-coupled receptors (MRGPRs), predominantly expressed on sensory neurons and immune cells, act as innate sensors mediating nociceptive sensations of pain and itch (65, 66). MRGPRX1, the first human MRGPR identified, is expressed predominantly on primary sensory neurons and represents a promising target for itch relief and pain suppression (67). Der p1, a cysteine protease and major allergen of house dust mites, induces the release of the pro-inflammatory cytokine IL-6, in part through activation of MRGPRX1, suggesting that MRGPRX1 is potentially involved in neuroinflammatory mechanisms in AR and allergic asthma. Thus, MRGPRX1 antagonists may hold therapeutic value in treating AR and allergic asthma (68).

Histamine, a pivotal mediator in AR (69) that mediates vasodilation, triggers responses through four histamine receptor (HR) subtypes identified in neurons: H1R, H2R, H3R and H4R. H1R is the principal functional receptor mediating histamine-induced responses in DRG neurons, leading to chronic itching and scratching behavior (70). Activation of H2R can induce airway mucus production, vascular permeability, and secretion of gastric acid. H3R plays an important role in neuro-inflammatory diseases. Furthermore, H4R has been demonstrated to be involved in allergy and inflammation (71). RNA-Seq has confirmed the presence of histamine receptors in TG neurons and that they are predominantly H1R and H3R (16). Studies have demonstrated that histamine can mediate the hypersensitivity response by inducing Ca^2+^ influx through interactions with H1R and TRPV1 on sensory neurons (72). Furthermore, nasal neurons release secretoneurin (SN) in response to histamine, promoting inflammatory cell recruitment in AR (73). Further investigation is required to elucidate the role of neuronal HR, in particular H2R, H3R and H4R, in allergic rhinitis.

## 4 Role of neuropeptide release from nociceptive neurons in the AR

Nociceptive stimuli activating receptors on nociceptive neurons induce depolarization and release of neuropeptides. These neuropeptides contribute to several pathological processes, including nociceptive transmission, neuronal survival, immune regulation, and the initiation of allergic and neurogenic inflammation. Elevated levels of neuropeptides such as SP and CGRP are observed in the airways of patients with allergic airway inflammation. Ablation or silencing of nociceptive neurons reduces neuropeptide expression and mitigates allergic airway inflammation (25, 52, 74). Neuropeptides act on blood vessels, smooth muscle and various immune cell populations, including dendritic cells (DCs), macrophages, MCs and innate lymphoid cells (11, 75–77), leading to oedema, vasodilatation, smooth muscle contraction and immune cell recruitment and activation. Moreover, the action of neuropeptides is limited by enzymatic degradation. Neutral endopeptidases on epithelial cell surfaces, glands and endothelium, play a significant role in limiting the extent and duration of neurogenic inflammation by breaking down neuropeptides (78). The role of neuropeptides in AR is shown in **Table 1**.

**Table 1:** The role of neuropeptides in allergic rhinitis.

Neuropeptide	Effect	Target	Cite
**SP**	promoting migration of human basophils	NK-1R in basophils	(79)
	Sch 50971 blocking substance P release:inhibiting nose rubbing behaviors and sneezing	—	(80)
	promoting nasal congestion;	direct neurokinin receptor activation independently of mast cell activation	(81, 82)
	substance P-mast cell interaction:promoting the mediator response to nasal allergen challenge	substance P-mast cell interaction (MRGPRX2 in mast cell)	
	promoting plasma extravasation and glandular secretion	—	(83, 84)
**CGRP**	promoting Vasodilation	CGRPR in blood vessels	(85)
	downregulation of type 2 cytokines and ILC2s	CGRPR in ILC2s	(86)
	inhibiting AHR	—	(87)
**VIP**	stimulating ILC2s and TH2 cells	VPAC2R in ILC2s and TH2 cells	(52)
**GAL**	GALR2 antagonist: inhibiting nose rubbing behaviors and sneezing	GALR2 in B cells	(88)
**TAFA4**	inhibiting the antigen-related mast cell activation	—	(89)
	inducing antigen-specific Tr1 cells;improving AIT’s therapeutic efficacy	FPR1 in DCs	(90)
**NMB**	mediating sneezing	central NMBR^+^ neurons in the sneeze-evoking region of the brainstem	(91)
**NMU**	activating ILC2s to produce type 2 cytokines	NMUR1 in ILC2s	(92, 93)

The symbol “–” indicates that the specific target is not stated in the corresponding reference.

### 4.1 SP

SP, a tachykinin family neuropeptide primarily secreted by nociceptive neuronal C fibers (78), functions by promoting vasodilation, increasing vascular permeability and stimulating secretion from nasal glands. Emerging evidence indicates SP’s immunomodulatory functions influence immune cells like B cells, neutrophils and DCs. SP receptors include mainly neurokinin-1 receptors (NK1-R) and MRGPRX2/B2. NK1R mediates the inflammatory response of the immune system and is expressed on basophils, eosinophils, neutrophils and others (94). MRGPRX2/B2, expressed mainly on mast cells but also on basophils, eosinophils and other cells (95). SP release from nociceptive neurons promotes the polarization of B cells into germinal center B cells, the release of antibody-secreting cells and the release of IgG and IgE. Nociceptive neurons and B cells participate in a feed-forward pro-inflammatory loop that amplifies adaptive immune responses (96). In addition to B cells, SP induces neutrophil chemotaxis and neutrophil-neuron cluster formation (25). After the release of SP from allergen-activated TRPV1^+^ nociceptive neurons, it modulates MRGPRA1 on CD301b^+^ DCs to induce cell migration and initiate adaptive type 2 immunity (97). Nociceptive sensory neurons also influence the inflammatory response in the AR by releasing SP that acts on NK1-R on basophils (79). Additionally, the released SP influences the symptoms of sneezing and nasal congestion present in AR. H3R, present on C fibers in the peripheral endings of sensory nerves, regulates SP release through ATP-sensitive K^+^ channels. Studies demonstrate that the H3R agonist Sch 50971 acts on the H3R to block SP release, thereby alleviating sneezing and nasal swabbing symptoms of AR (80). SP-induced exacerbation of nasal congestion is also mediated by direct activation of neurokinin receptors independent of MCs activation. However, during allergic reactions, SP interacts with MCs to enhance inflammatory mediators in response to allergen-stimulated nasal responses (81), which may be related to MRGPRX2/B2. In addition to the classical receptor NK-1R, SP activates human mast cells to release a variety of pro-inflammatory cytokines and chemokines through the activation of MRGPRX2 (98). SP activates MRGPRX2 on mast cells, induces histamine release and can lead to allergic airway inflammation (82). However, the specific mechanisms by which MRGPRX2 affects allergic rhinitis remain to be elucidated. Furthermore, the TRPV1-SP axis plays a vital role in the pathogenesis of AR. SP co-localizes with TRPV1 on trigeminal nerve-nociceptive neurons in the mouse nasal mucosa. Activation of TRPV1 on nociceptive neurons triggers SP-mediated neurogenic inflammation (83). Intranasal needling as a nociceptive stimulus to the nasal mucosa activates TRPV1, causing nasal mucosal sensory nerve fibers to secrete large amounts of SP. Interestingly, as the intensity of needling increases, there is a decrease in TRPV1 activity, a depletion of SP and a dulling and degeneration of nerve fibers (84). Thus, these findings suggest that intranasal needling could provide a potential treatment avenue for AR by decreasing TRPV1 activity, consequently reducing the density and number of SP nerve fibers.

### 4.2 CGRP

CGRP, primarily released from activated trigeminal afferent fibers, acts as a nociceptive mediator when reaching pathological concentrations (42). CGRP-containing nerve fibers are abundant in the human nasal mucosa, mainly localized around blood vessels and glands (99). CGRP is comprised of two isoforms, alpha and beta. CGRPα is primarily expressed in the central and peripheral nervous systems, while CGRPβ is predominantly expressed in the enteric nervous system (100). CGRP receptors, predominantly on blood vessels, especially arterioles (78), contribute to potent vasodilatory effects (85). Increases in CGRP^+^ neurons are observed in airway inflammatory responses (101). Moreover, CGRP has been demonstrated to stimulate the activation of the cAMP - PKA axis and inhibit pro-inflammatory cytokine production through inducible cAMP early repressor (ICER)-dependent transcriptional repression (102). Additionally, CGRP, a critical negative regulator of the group 2 innate lymphoid cell (ILC2) response *in vivo*, can effectively suppress airway inflammation (86). A recent study has demonstrated that this inhibition is primarily mediated by this isoform of CGRPβ (103). In sensitized mice, allergen provocation induced eosinophilic airway inflammation and allergic airway hyperresponsiveness, resulting in a significant reduction of CGRP in neuroepithelial cell bodies and submucosal plexus, whereas CGRP administration into sensitized mice normalized airway hyperresponsiveness (87). Recent clinical evidence supports the use of noninhaled intranasal delivery of 100% CO2 for treatment of allergic rhinitis (104). CGRP also co-localized with TRPV1^+^ nociceptive neurons, and intranasal delivery of 100% CO_2_ inhibited the activating effect of capsaicin on nociceptive neurons and reduced CGRP release, ameliorating AR symptoms (105). Thus, CGRP may exert both pro-inflammatory and anti-inflammatory effects. CGRP mediates neurogenic inflammation, dilates blood vessels and obstructs the nasal passages, causing nasal congestion and blocked nose. However, it may also act as an anti-inflammatory mediator to inhibit pro-inflammatory cytokine production and negatively regulate ILC2, suppressing airway inflammation and alleviating allergic airway hyperresponsiveness. Nevertheless, the CGRP release from nociceptive neurons in AR is a complex mechanism, warranting further study.

### 4.3 Others: VIP, GAL, TAFA4, NMB, NMU

Apart from the central neuropeptides, SP and CGRP, several other neuropeptides also contribute to the pathogenesis of AR. A vasoactive intestinal polypeptide (VIP), primarily derived from parasympathetic nerves, has recently been identified as originating from Nav1.8^+^ nociceptive neurons in both the central and peripheral nervous system (11, 52). It plays a role in vasodilation. The nasal mucosal tissue of patients with AR exhibits significantly more VIP-positive nerve fibers than controls (99, 106). In allergic airway inflammation, IL-5 stimulates nociceptive neurons to release VIP, which further activates ILC2 and Th2 cells via VPAC2R, intensifying the airway inflammatory response (52). Galanin (GAL) is a widely expressed neuropeptide and plays essential roles in nociception perception, synaptic transmission and inflammatory responses (107). A study demonstrated that nose-swabbing behavior and sneezing were significantly reduced in AR mice after using a galanin receptor 2 (GALR2) inhibitor, indicating a potential role of GAL-GALR2 signaling in AR development (88). Derived from the C-fiber mechanically nociceptive receptors, TAFA chemokine-like family member 4 (TAFA4) is a neuropeptide that has immunomodulatory functions. It downregulates the expression of mast cell FcϵRI by activating the PTEN-PU.1 pathway and reduces allergic mediators such as mast cell protease 1 (MCPT-1) and eosinophil peroxidase (EPX), as well as Th2 cytokine (IL-4, IL-5, IL-13) levels to alleviate AR symptoms (89). TAFA4 also induces the release of IL-10 from DCs by activating the FPR1-MyD88-AKT signaling pathway, attenuating the inflammatory response, and relieving allergic symptoms in AR mice. Additionally, TAFA4 can be used to enhance the therapeutic effect of allergen-specific immunotherapy (AIT) on AR by inducing the expression of antigen-specific Type 1 regulatory T (Tr1) cells (90). Neuromedin B (NMB) regulates the perception of nociception in sensory neurons. Recent studies have reported that allergy-induced sneezing behavior is closely related to NMB. Nociceptive neurons in the nasal mucosa receive external stimuli, consequently releasing NMB and triggering sneezing. Furthermore, the NMB activates NMBR^+^ neurons in the brainstem that mediate the sneeze reflex, which ultimately projects to the caudal ventral respiratory group (cVRG) and leads to sneezing (91). NMU, a highly conserved multifunctional neuropeptide, is often considered to be secreted by cholinergic neurons (108). However, some studies have found that sensory neurons innervating the airways express NMU (109). A recent study has demonstrated that sensory neuron-derived NMU plays a role in the pathogenesis of allergic airway inflammation, acting via the NMU/NMUR1 axis to activate ILC2 (92). Furthermore, it has been demonstrated that NMU induces the activation of peripheral ILC2 through the ERK pathway in allergic rhinitis (93). In addition to NMUR1, NMU directly induces degranulation of skin mast cells, presumably via MRGPRX2. The role of NMU-MRGPRX2/MrgprB2 signaling in mast cells in allergic rhinitis may be a promising avenue for further investigation (110).

## 5 Immune system activates nociceptive neurons

Nociceptive neurons sense nociceptive stimuli and release neuropeptides that affect the immune system. Immune cells and inflammatory mediators can increase the sensitivity of nociceptive neurons to nasal stimuli, mediating depolarization or directly activating nociceptive neurons, thereby lowering the threshold for action potential generation (11, 111). In AR, the modulatory effects of inflammatory responses on nociceptive neurons have been observed. Recent studies show that patients with AR exhibit higher trigeminal nerve sensitivity compared to healthy controls, potentially linked to inflammatory changes in AR (112). The role of the immune system on nociceptive neurons is shown in **Table 2**.

**Table 2:** The role of the immune system on nociceptive neurons.

Inflammatory mediator	Immune cells that release inflammatory mediators	Target	Effect	Cite
histamine	mast cell;basophil	HR:H1R, H3R	promoting nasal pruritus; promoting sneezing; Brain activation provocation	(113–115)
PGD2	mast cell	DP1	reducing activation threshold of nasal sensory neurons	(116)
tryptase	mast cell	PAR2	upregulating neurogenic inflammation; upregulating Bradykinin	(85, 117)
Leukotriene D4	basophil	CysLTR1	increasing the excitability of nasal nociceptive sensory neurones	(118)
NGF	eosinophil;mast cell;basophil	TrkA;p75	increasing expression of voltage-gated sodium channels (NaV1.7, NaV1.8 and NaV1.9); promoting BDNF release, synergistically sensitising nociceptive neurons; increasing nasal sensitivity, airway reactivity, and neuronal hypersensitivity	(20, 119–122)
NT-3	eosinophil	TrkC	affect the nasal mucosa locally	(123)
TNF-α	macrophage;monocyte	TNFR	reducing activation threshold of nociceptive neurons; increasing AHR	(9, 124)
IL-4	Th2	IL-4R	increasing the sensitivity of nociceptive neurons	(125)
IgE	plasma cells	FcϵR1	activating nociceptive neurons;initiating and amplifying allergic airway inflammation	(64)

### 5.1 Immune cells and inflammatory mediators activate nociceptive neurons

Immune cells release inflammatory mediators to alter the sensitivity of nociceptive neurons and participate in the regulation of itching and pain (24). For example, MCs and basophils, activated during nasal allergen deposition in patients with AR, release histamine. Histamine activates the H1R on trigeminal neuron C fibers, which transmits the sensation of itching (113). In addition to H1R, histamine also exacerbates certain symptoms of AR, such as sneezing, by acting on neuronal H3R in the caudal part of the nucleus caudalis of the dorsal trigeminal spinal cord in the brainstem (114). Furthermore, histamine, through the activation of nasal sensory nerve endings and upward conduction to the CNS, triggers the activation of brain regions related to psychoemotional changes and even neurological symptoms in patients with AR (115). Leukotriene D4 (LTD4), a nasal mucosal vasodilator (126), secreted mainly by basophils. LTD4 has been demonstrated to directly increase the excitability of guinea pig trigeminal neurons via cysteinyl leukotriene receptor 1 (CysLTR1) (118). Furthermore, it has been demonstrated that Leukotriene C4 (LTC4), released by basophils, acts on CysLTR2 on sensory neurons to mediate acute pruritus (127). Nevertheless, CysLTR2 expression has not been identified on nasal nociceptive neurons (118). The precise role of basophil-derived LTC4-CysLTR2 in allergic rhinitis remains to be elucidated. Prostaglandins and tryptase released by MCs can also activate and sensitize nociceptive neurons. Studies also report that the binding of tryptase, released by MCs, with proteinase-activated receptor 2 (PAR2) on airway-nociceptive neurons leads to the release of SP and CGRP and promotes airway neurogenic inflammation (117). Tryptase also break down kininogen in the blood, producing the inflammatory mediator kinin (128). Similarly, BK acts directly on vascular bradykinin receptors to promote vascular permeability, and it also stimulates nociceptive sensory nerves and transmits nociceptive signals to preganglionic autonomic neurons in the brainstem, thereby eliciting parasympathetic reflexes and inducing glandular secretion. Notably, a significant increase in responsiveness to BK has been reported in patients with severe AR (85). PGD2, a major cyclooxygenase metabolite, acts on prostaglandin D2 receptor 1 (DP1), lowering the action potential threshold of small-diameter neurons to modulate neuronal excitation induced by various stimuli. Furthermore, the activation of PGD2-DP1 receptor signaling leads to a significant increase in histamine-induced action potentials and depolarizations, thereby exacerbating rhinitis symptoms in guinea pigs (116). Moreover, PGD2 dilate vessels in the nasal mucosa, resulting in nasal congestion (126). It has also been shown that prostanoids such as Prostaglandin E2 (PGE2) and Leukotriene B4 (LTB4) do not directly stimulate nociceptive neurons but rather make neurons more susceptible to depolarization by altering the threshold for depolarization (8).

In addition to MCs and basophils, many other immune cells and related inflammatory mediators are involved in activating nociceptive neurons. A close relationship exists between plasma cells and nociceptive neurons. After OVA sensitization, plasma cells have been reported to secrete IgE to form immune complexes with OVA, which regulates the excitability of nociceptive neurons and enhances allergic airway inflammation (64). In response to inflammation, neutrophils produce direct-acting mediators, such as PGE2, which contribute to hypernociception (129). Furthermore, activated eosinophil products also increase afferent nerve excitability at sites of allergic inflammation. Eosinophils, isolated from the peripheral blood of patients with AR, were found to activate DRG neurons cultured *in vitro (* 130).

Monocytes and macrophages also play an essential role in the nociceptive sensation of allergic diseases. These cells also act directly on nociceptive neurons by releasing inflammatory mediators such as tumor Necrosis Factor α (TNF-α), enhancing neuronal excitability by modulating receptors such as TRPA1, TRPV1 and Nav1.7-1.9 affecting nociceptive neurons (24, 74, 131). TNF-α plays an important role in allergic diseases as a pro-inflammatory cytokine. TG neuronal cell bodies are surrounded by a specialized type of glial cells called satellite glial cells, which communicate directly through gap junctions and are involved in sensitizing TG-nociceptive neurons. Notably, TNF-α expression levels are elevated in the nasal secretions of patients with AR (132). TNF-α increases the levels of connexin 26 and activates p38 mitogen-activated protein kinase in the TG, augmenting the gap junction activity, promoting neuron-satellite glial cell communication and reducing the activation threshold required for nociceptive neurons (124). TNF-α activates TRPV1^+^ DRG neurons, sensitizing them to TRPV1 activators, leading to airway hyperresponsiveness (9). Th2 cytokines, such as IL-4, are also associated with neuronal hyperresponsiveness in allergic inflammation. In a mouse model of allergic airway inflammation, immune cells were found to act on nociceptive neurons by releasing IL-4 and IL-13, altering the transcriptome of nociceptive neurons during type 2 inflammation, overexpressing neuronal sensitivity-related genes and enhancing the sensitivity of nociceptive neurons to nociceptive stimuli (125).

### 5.2 Role of neurotrophic factors in nociceptive neurons

The neurotrophic factor family comprises NGF, brain-derived neurotrophic factor (BDNF), neurotrophin-3 (NT-3) and neurotrophin-4/5 (NT-4/5). Various immune cells, such as MCs, eosinophils and macrophages, release these neurotrophic factors. Neurotrophic factors are essential for neuronal development, modulating excitability in mature neurons and playing a role in neurogenic inflammation (7, 120). Some studies suggest that early-life allergic inflammation may lead to persistent abnormalities in nociceptive neural circuits, impacting sensitivity to external stimuli during a critical development ‘window period’ (6).

NGF, which is critical for the development of nociceptive C fibers, induces biochemical and structural changes in nerves, influencing the expression of sensory neuropeptides and TRPV1, and contributing to hyperresponsiveness. NGF receptors, TrkA and p75, are present on sensory nerves in the human nasal mucosa. In patients with AR, baseline NGF levels in nasal lavage fluid are significantly higher than in healthy controls. After allergen stimulation, NGF levels and the number of NGF-positive nerve bundles increase rapidly in patients with AR. NGF acts on receptors in nociceptive neurons, affecting structural and biochemical processes, with NGF expression in the nasal epithelium positively correlating with neuronal marker PGP 9.5 and C-fiber density, demonstrating the effect of NGF on neuronal plasticity (121, 122). NGF also promoted BDNF release, synergistically sensitizing nociceptive neurons (120). Elevated NGF levels in AR contribute to increased expression of voltage-gated sodium channels (NaV1.7, NaV1.8 and NaV1.9), resulting in hypersensitivity (20). Additionally, local production of NT-3 in nasal mucosa may show their effects on the local site without joining the systemic circulation in AR patients, playing a role in AR neuronal inflammation (123). This effect may be attributed to the specific binding of NT-3 to TrkC receptors (133).

## 6 Conclusion

This review focuses on the pivotal role of nociceptive neurons in the nasal mucosa in AR pathogenesis (**Figure 1**). Nasal mucosal nociceptive neurons release neuropeptides, initiating a neuroimmune loop where immune cells release cytokines and other substances, which in turn, affect nociceptive neurons. Peripheral sensitization in AR may result from this intricate interaction. Nerve blockade therapy has shown efficacy in treating patients with AR insensitive to conventional treatments and immunotherapy (134, 135). Further exploration of the interaction between immune cells, inflammatory mediators and nociceptive neurons is crucial for developing neuro-targeted treatment of AR.
